# 

**Published:** 1908-11

**Authors:** 


**CONTENTS—NOVEMBER, 1908.—VOL. XXIV, NO. 5.**

ORIGINAL ARTICLES—

The Regulation of the Social Evil in Our Large American Cities. By Frank G. Lydston, M. D., Chicago .................... 183

EDITORIAL DEPARTMENT—

Regulating Professional Conduct by Law .................... 191
Belling the Cat .................... 196
The Trustees Can't See It .................... 202

EDITORIALETS .................... 203-204

COMMUNICATIONS .................... 204-207

ABSTRACTS AND SELECTIONS .................... 207-216

BOOKS AND MAGAZINES .................... 216-223

PUBLISHER'S DEPARTMENT .................... 223-228

